# Effects of serum N-terminal pro B-type natriuretic peptide and D-dimer levels on patients with acute ischemic stroke

**DOI:** 10.12669/pjms.344.15432

**Published:** 2018

**Authors:** Jia Li, Chengzhi Gu, Dan Li, Lan Chen, Zhenhui Lu, Lianhai Zhu, Huaiyu Huang

**Affiliations:** 1 Jia Li, Department of Neurology, Second Affiliated Hospital of Nantong University, Nantong 226001, Jiangsu Province, China; 2 Chengzhi Gu, Department of Neurology, Second Affiliated Hospital of Nantong University, Nantong 226001, Jiangsu Province, China; 3 Dan Li, Department of Neurology, Second Affiliated Hospital of Nantong University, Nantong 226001, Jiangsu Province, China; 4 Lan Chen, Department of Neurology, Second Affiliated Hospital of Nantong University, Nantong 226001, Jiangsu Province, China; 5 Zhenhui Lu, Department of Neurology, Second Affiliated Hospital of Nantong University, Nantong 226001, Jiangsu Province, China; 6 Lianhai Zhu, Department of Neurology, Second Affiliated Hospital of Nantong University, Nantong 226001, Jiangsu Province, China; 7 Huaiyu Huang, Department of Neurology, Second Affiliated Hospital of Nantong University, Nantong 226001, Jiangsu Province, China

**Keywords:** Acute ischemic stroke, D-dimer, N-terminal pro B-type natriuretic peptide

## Abstract

**Objective:**

To detect the serum levels of D-dimer and N-terminal pro B-type natriuretic peptide (NT-pro BNP) in patients with Acute Ischemic Stroke (AIS), and to explore the risk factors of AIS.

**Methods:**

A total of 246 AIS patients treated in our hospital from January 2015 to January 2017 were selected. Meanwhile, 240 healthy subjects were selected as a control group. The D-dimer and NT-pro BNP levels of the two groups were compared. Correlations of such levels with age, gender, blood lipid, Intima-Media Thickness (IMT), fibrinogen and degree of neurological deficits were analyzed.

**Results:**

The AIS group had significantly higher levels of Triglyceride (TG), Low-Density Lipoprotein (LDL), D-dimer, NT-pro BNP and fibrinogen as well as IMT than those of the control group, but the High-Density Lipoprotein (HDL) level of the AIS group was significantly lower (P<0.05). The patients with different genders and ages had significantly different D-dimer and NT-pro BNP levels (P<0.05). The D-dimer and NT-pro BNP levels were correlated with gender and age. Such levels of females were significantly higher than those of males (P<0.05). The D-dimer and NT-pro BNP levels of the ≥60 years old group significantly exceeded those of the <60 years old group (P<0.05). The levels of D-dimer and NT-pro BNP were negatively correlated with that of HDL (P<0.05), but positively correlated with TG, LDL and fibrinogen levels, IMT, and National Institutes of Health Stroke Scale score (P<0.05). Multivariate Logistic regression analysis showed that the OR values of D-dimer and NT-pro BNP were 3.65 and 6.96 respectively.

**Conclusion:**

Serum D-dimer and NT-pro BNP levels usually increased in AIS patients, and the levels were significantly correlated with AIS onset.

## INTRODUCTION

Stroke is a common clinical disease, including ischemic and hemorrhagic types. Its high morbidity and disability rates easily lead to death of patients in serious cases, exerting a certain impact on human health and quality of life, especially those of the elderly. At present, about 73.7-751/100,000 people are stricken with stroke each year in China, and the death rate reaches 40.5-98.5/100,000. As a result, the life span of residents is affected, also involving many young people.^1^

Acute Ischemic Stroke (AIS) is associated with impaired blood supply to the brain, leading to ischemia and hypoxia in brain tissues. In severe cases, brain tissues are irreversibly damaged.^2^ Generally, AIS patients are complicated with neurological damages after onset. AIS includes both acute and convalescent phases, of which the acute phase mainly refers to the period within two weeks after onset. Such patients may have more obvious pathological and physiological changes, and unstable conditions. With elapsed time, their AIS symptoms are gradually relieved, entering the convalescent phase.^3^,^4^

B-type Natriuretic Peptide (BNP) is related with brain tissue injury. It originates from brain tissues and myocardial cells, and is associated with blood pressure regulation and balance between water and sodium.^5^,^6^ When tissues are damaged, BNP precursor enters the blood, and decomposes into N-terminal pro BNP (NT-pro BNP) and BNP. Particularly, NT-pro BNP has a longer half-life than that of BNP, and is more sensitive to changes. Therefore, NT-pro BNP is commonly selected to assess the degree of injury and prognosis of patients. When brain tissues are injured, the serum NT-pro BNP level rises, especially in stroke patients.^7^,^8^ As the damage is aggravated, the level further increases. Thus, it can be used to evaluate the progression of stroke and the severity of neurological deficits. Plasma D-dimer, as the end hydrolysate of plasma fibrous protein, is associated with thrombosis, decomposition and fibrinolytic level.

In this study, the serum levels of D-dimer and NT-pro BNP in AIS patients were detected, and the relationship between them was analyzed. Correlation analysis was performed according to the scores of National Institutes of Health Stroke Scale (NIHSS), aiming to provide a clinical reference for the prevention, treatment and prognosis evaluation of AIS.

## METHODS

A total of 246 AIS patients treated in our hospital from January 2015 to January 2017 were selected, all of whom met the diagnostic criteria of AIS. AIS was confirmed by CT and MRI, with the onset within 72 hour. There were 130 males and 116 females aged between 57 and 79 years old, with an average of (64.2 ± 4.8). Patients with tumors, infections, immune diseases, or abnormal heart, liver and kidney functions were excluded. In the meantime, 240 healthy subjects were selected as a control group, including 122 males and 118 females aged between 56 and 79 years old, with an average of (64.3 ± 4.5). The two groups had similar baseline clinical data (P>0.05). This study has been approved by the ethics committee of our hospital. Before treatment, the family members of all patients were informed and signed the written informed consent.

### Ultrasonography of Carotid Artery

The Intima-Media Thickness (IMT) of carotid artery was measured by GE Vivid7 color Doppler ultrasound diagnostic apparatus, with the probe frequency of 7.5-10.0 MHz. All examinations were performed by the same medical technician. Patient took a supine position without pillow, and the examination site was deflected to the contralateral side through the head to expose the neck, and bilateral common carotid arteries, carotid bifurcation, internal carotid artery and external carotid artery were carefully explored along the outer edge of the lateral and longitudinal directions through the sternocleidomastoid muscle. Normal: IMT <1.0 mm; carotid atherosclerosis: IMT = 1-1.2 mm; plaque: IMT >1.2 mm.

### Sample Collection

In the early morning, 3 ml of fasting elbow venous blood was collected from all patients and healthy subjects, and then left still for 30 minutes. Afterwards, serum was separated by centrifugation at 3000 rpm for 10 min and stored in a -20°C refrigerator prior to use.

### Detection of Biochemical Indices

The double antibody sandwich assay was used to detect D-dimer levels, and the immunofluorescence assay was used to measure NT-pro BNP levels. HITACHI 7600 automatic biochemical analyzer was used to detect the levels of Total Cholesterol (TC), High Density Lipoprotein (HDL), Low Density Lipoprotein (LDL), Triglyceride (TG) and fibrinogen, together with platelet and leukocyte counts.

### Scoring of Neurological Deficits and Prognosis

NIHSS was used to evaluate the conditions of patients at admission. NIHSS score: Mild neurological dysfunction: ≤7 points; severe neurological dysfunction: >7 points.

### Statistical Analysis

All data were analyzed by SPSS 16.0 software. The categorical data were expressed as mean ± standard deviation (x ± s). Comparisons among multiple groups were performed by one-way analysis of variance, and pairwise comparisons were conducted by the SNK-q test. The numerical data were expressed as percentage and subjected to the χ^2^ test. Pearson’s correlation analysis was employed. Correlations of D-dimer and NT-pro BNP levels with AIS were studied by multivariate Logistic regression analysis. P<0.05 was considered statistically significant.

## RESULTS

### AIS-related Risk Factors

Significantly more cases in the AIS group had histories of hypertension, diabetes, smoking and alcohol drinking than those of the control group (P<0.05). The two groups had similar CHOL levels (P>0.05). The AIS group had significantly higher levels of TG, LDL, D-dimer, NT-pro BNP and fibrinogen as well as IMT than those of the control group, but the HDL level of the AIS group was significantly lower (P<0.05) (Table-I).

**Table-I:** AIS-related risk factors.

Index	AIS group (n=246)	Control group (n=240)	χ^2^/t	P
Hypertension	130 (52.8)	8 (3.3)	146.470	0.000
Diabetes	64 (26.0)	4 (1.7)	59.852	0.000
Smoking	128 (52.0)	30 (12.5)	86.529	0.000
Alcohol drinking	100 (40.7)	42 (17.5)	31.481	0.000
TG (nmol/L)	2.05±0.87	1.42±0.73	8.637	0.000
CHOL (nmol/L)	5.21±1.09	4.87±0.88	3.778	0.000
HDL (nmol/L)	1.09±0.23	1.25±0.36	5.853	0.000
LDL (nmol/L)	3.33±0.81	2.79±0.81	7.348	0.000
IMT (mm)	1.07±0.33	0.48±0.13	25.812	0.000
Fibrinogen (g/L)	5.72±1.04	2.43±0.39	45.956	0.000
D-dimer (mg/L)	1.18±0.28	0.35±0.17	39.383	0.000
NT-pro BNP (pg/mL)	625.25±59.83	58.62±7.45	145.618	0.000

### D-dimer and NT-pro BNP levels of patients of different genders and ages

The D-dimer and NT-pro BNP levels of females were significantly higher than those of males (P<0.05). The D-dimer and NT-pro BNP levels of the ≥60 years old group significantly exceeded those of the <60 years old group (P<0.05) (Table-II).

**Table-II:** D-dimer and NT-pro BNP levels of patients of different genders and ages

Factor		n	D-dimer (mg/L)	NT-pro BNP (pg/mL)
Gender	Male	130	0.95±0.24	601.23±52.37
	Female	116	1.25±0.31	662.17±61.39
	t		8.534	8.400
	P		0.000	0.000
Age (year)	≥60	142	1.26±0.31	638.19±52.88
	<60	104	0.91±0.25	603.37±62.18
	t		9.475	4.734
	P		0.000	0.000

### Correlations between D-dimer, NT-pro BNP levels and other indices

The levels of D-dimer and NT-pro BNP were negatively correlated with that of HDL (P<0.05), but positively correlated with TG, LDL and fibrinogen levels, IMT, and NIHSS score (P<0.05) (Table-III).

**Table-III:** Correlations between D-dimer, NT-pro BNP levels and other indices

Index	D-dimer		NT-pro BNP	

	r	P	r	P
Gender	0.159	0.042	0.258	0.041
Age (year)	0.215	0.042	0.273	0.036
D-dimer	-	-	0.306	0.038
NT-pro BNP	0.306	0.038	-	-
TG	0.339	0.041	0.248	0.047
CHOL	0.041	0.487	0.021	0.758
HDL	-0.321	0.038	-0.498	0.031
LDL	0.318	0.044	0.387	0.041
IMT	0.312	0.031	0.287	0.038
Fibrinogen	0.268	0.038	0.238	0.041
NIHSS score	0.696	0.024	0.714	0.021

### Correlations between D-dimer, NT-pro BNP levels and AIS

Multivariate Logistic regression analysis showed that the OR and 95%CI values of D-dimer and NT-pro BNP were 3.65 (1.38-10.92) and 6.96 (1.98-11.27) respectively (P<0.05) (Table-IV). Therefore, there were significant correlations between D-dimer, NT-pro BNP levels and AIS.

**Table-IV:** Correlations between D-dimer, NT-pro BNP levels and AIS.

	Variable	β	Wals	P	OR (95%CI)
Univariate	D-dimer	1.275	7.838	0.004	3.31 (1.47-6.98)
	NT-pro BNP	1.438	11.265	0.001	6.87 (2.01-9.28)
Logistic	D-dimer	1.312	6.468	0.012	3.65 (1.38-10.92)
	NT-pro BNP	1.498	10.154	0.016	6.96 (1.98-11.27)

## DISCUSSION

AIS has a high incidence and mortality, with complicated pathogenesis. D-dimer has a certain relation with the occurrence of atherosclerosis and cerebral infarction.^9^ A higher level of D-dimer is easily accumulated in the blood vessel wall, which can promote platelet adhesion and atherosclerosis, and further induce the formation of thrombus. The level of D-dimer increases significantly when the blood coagulation and fibrinolysis system in patients with cerebrovascular diseases is out of balance and maintains an activated state.^10^ Studies have shown that D-dimer levels in patients with plaques in the carotid arteries are significantly higher than the levels in those without plaques,^11^,^12^ suggesting that D-dimer can cooperate with the coagulation system to participate in the occurrence and progression of atherosclerosis. Therefore, patients with higher D-dimer levels need clinical intervention and treatment. B-type brain natriuretic peptide exists in brain tissue and myocardial tissue, and has a sensitive prediction function for the degree of heart failure. When brain tissue damage occurs, NT-pro BNP levels increase, and the neurological deficits will be more serious, which is related to the clinical diagnosis, treatment, disease grading, and prognosis of patients with AIS.^13^ This study shows that there is a correlation between D-dimer, NT-pro BNP, and gender and age. Females have higher levels of D-dimer and NT-pro BNP than men. And the D-dimer and NT-pro BNP levels of the elderly are higher than those of young people. In the AIS group, D-dimer and NT-pro BNP levels were positively correlated with LDL and TG (P<0.05), which may be due to the influence of D-dimer on the synthesis and secretion of lipid apolipoproteins Apo B and Lp(a). In addition, Apo B and Lp(a) are involved in the synthesis and assembly of VLDL, LDL, and IDL, and different levels of Apo B and Lp(a) can bind to fibrin binding sites, inhibit the activity of plasmin, and lead to the formation of thrombus, with compensatory secondary hyperfibrinolysis, and cause a significant increase in D-dimer levels.^14^ Different levels of VLDL have a certain impact on the body’s fibrinolytic function, and can promote the formation of thrombosis.^15^ Serious thrombosis can lead to severe secondary hyperfibrinolysis. And after entering into the liver, VLDL can be converted into LDL through metabolism, which can affect LDL levels and indirectly the body’s D-dimer levels. Endothelial cells contain a large number of neutral endopeptidases, and abnormal blood lipid levels can damage the body’s vascular endothelial cells, leading to a large number of neutral endopeptidases released from endothelial cells. BNP levels increase with BNP hydrolysis, resulting in the increase in blood NT-pro BNP levels.

D-dimer is associated with the body’s thrombin activity and can reflect the degree of thrombosis or dissolution in patients. Excessively high D-dimer accumulated in the blood vessel wall will directly damage the blood vessel, resulting in the occurrence of endothelial dysfunction. In severe cases, atherosclerosis may occur, and platelets may adhere and accumulate to maintain the high coagulation state of blood.^16^ The results of this study indicate that the level of D-dimer in patients with AIS is significantly higher than that in the control group, and there is a significant positive correlation with IMT and fibrinogen in patients, suggesting that coagulation in patients with AIS. Higher levels of D-dimer can inhibit thrombin activity, promote thrombus enlargement and progression, and aggravate neurological deficits in patients. Atherosclerosis is associated with the occurrence of cardiovascular and cerebrovascular diseases. The literature indicates that the progressive plaques in the coronary artery are accompanied with the expression of some BNPs, and can reduce the proliferation and migration of smooth muscle cells through binding to NPR-A.^17^ This study showed that NT-pro BNP levels in patients with AIS were positively correlated with IMT and NIHSS scores (P<0.05), indicating that the occurrence of atherosclerosis is also a risk factor for the increase of NT-pro BNP levels, and at the same time increases the risk of the incidence of ischemia stroke. Fibrinogen can change blood components, leading to abnormal blood flow and further damage the blood vessel wall, which can indirectly cause thrombosis. NT-pro BNP can regulate the function of the cardiovascular system. Thrombosis can accelerate the secretion of NT-pro BNP, which in turn speeds up the loss of endothelial function, leading to concentrated blood concentration and further increase in blood viscosity. This indirectly promotes the body’s thrombosis, and also suggests that NT-pro BNP has a synergistic effect on the formation of fibrinogen-induced thrombosis.

In summary, patients with AIS are usually accompanied by increased levels of serum D-dimer and NT-pro BNP, and such levels are significantly correlated with AIS.

### Authors’ contributions

**JL, CG & HH:** Designed this study and significantly revised this manuscript.

**DL, LC, ZL & LZ:** Performed this study and analyzed clinical data.
